# Primary mucinous carcinoma of the skin with co-expression of TRPS1 and GATA3: a case report

**DOI:** 10.3389/fonc.2025.1530871

**Published:** 2025-09-03

**Authors:** Liling Song, Ning Zhu, Lei Jiang, Dong Gao, Guohua Yu

**Affiliations:** ^1^ The Second School of Clinical Medicine of Binzhou Medical University, Yantai, Shandong, China; ^2^ Department of Pathology, Yantai Yuhuangding Hospital, The Affiliated Hospital of Qingdao University, Yantai, China; ^3^ Department of Pathology, College of Basic Medical Sciences, Binzhou Medical University, Yantai, Shandong, China; ^4^ Department of Dermatology, Laishan Branch of Yantai Yuhuangding Hospital, The Affiliated Hospital of Qingdao University, Yantai, China

**Keywords:** PCMC, TRPS1, GATA3, co-expression, differential diagnosis

## Abstract

Primary cutaneous mucinous carcinoma (PCMC) is a rare malignant neoplasm, with approximately 450 cases reported worldwide to date. Its histological features closely resemble those of mucinous carcinoma of the breast, posing significant diagnostic challenges. We report a case of PCMC occurring at the upper margin of the eyelid in a 65-year-old male who presented with a painless, progressively enlarging mass over a four-year period. Histopathological examination following surgical excision confirmed the diagnosis of PCMC, with immunohistochemical staining demonstrating co-expression of TRPS1 and GATA3. This case highlights several key clinical and pathological characteristics of PCMC. The tumor typically affects middle-aged to elderly males and demonstrates low metastatic potential but a high rate of local recurrence. Histologically, it is difficult to distinguish from cutaneous metastatic mucinous carcinoma (MMC), particularly of breast origin. In this context, the co-expression of TRPS1 and GATA3 necessitates careful interpretation, as these markers are not exclusive to PCMC. The diagnostic utility of TRPS1 lies not in its individual specificity, but rather in its combined use with other markers such as GATA3. Radical surgical excision remains the mainstay of treatment, with adjuvant endocrine therapy considered in ER/PR-positive cases. Compared to cutaneous MMC, PCMC generally carries a more favorable prognosis, reinforcing the importance of early and accurate diagnosis. Ultimately, the diagnosis of PCMC relies on a comprehensive evaluation that integrates clinical presentation, imaging findings, histological architecture, and immunohistochemical profiles.

## Introduction

PCMC is a rare malignant adnexal neoplasm that originates from eccrine or, more likely, apocrine glands (1, 2). It most frequently arises in apocrine-rich regions, particularly the head and neck area, and exhibits indolent behavior characterized by low metastatic potential but a high rate of local recurrence (3–7). Despite its slow growth, PCMC poses significant diagnostic challenges due to its histological resemblance to metastatic mucinous carcinoma (MMC), especially breast-derived variants (8–10).

Histologically, PCMC is composed of nests and clusters of epithelial cells suspended in abundant extracellular mucin, often mimicking the morphology of mucinous breast carcinoma (MBC) (8). This significant morphological overlap necessitates meticulous clinicopathological correlation and a comprehensive immunohistochemical workup, as conventional markers such as CK7, ER, PR, and GATA3 lack specificity due to their frequent expression in breast carcinomas (5, 10–12). Recent studies have identified TRPS1, a transcription factor involved in breast glandular differentiation, as a potentially useful marker in distinguishing PCMC. However, TRPS1 is not entirely mammary-specific and can be expressed in various adnexal and neuroendocrine neoplasms, further complicating the diagnostic landscape (13–17).

In this study, we present a rare case of PCMC located on the upper eyelid margin of a 65-year-old male, demonstrating co-expression of TRPS1 and GATA3, along with positivity for ER, PR, and WT1, and focal neuroendocrine marker expression. We aim to emphasize the diagnostic nuances associated with PCMC, particularly in differentiating it from cutaneous metastases, and to highlight the potential utility and limitations of TRPS1 in combination with other markers. Through this case, we seek to enhance pathologists’ awareness of this rare entity and contribute to the understanding of its immunophenotypic and molecular profile.

## Case presentation

A 65-year-old male patient first noticed a “mass on the upper margin of the left eyelid” four years ago (in November 2020). Over the past three months, the mass had gradually grown to the size of a soybean, causing no pain or itchiness. He subsequently visited our hospital. Specialty examination revealed a purplish-red mass with a diameter of approximately 2 cm on the upper margin of the left eyelid, protruding from the skin surface, partially ulcerated, and with unclear boundaries with surrounding tissues. The patient had previously been in good health, denied a family history of genetic diseases, and no similar diseases in his family. He also denied a history of exposure to radioactive materials. Physical examinations showed no obvious abnormalities in other systems. Imaging examinations of other systemic organs revealed no significant space-occupying lesions, and laboratory tests showed no abnormalities. The patient was admitted to the hospital for excision of the mass under the preliminary diagnosis of “mass on the upper margin of the left eyelid,” and the postoperative specimen was routinely sent for pathological examination.

**Figure 1:**
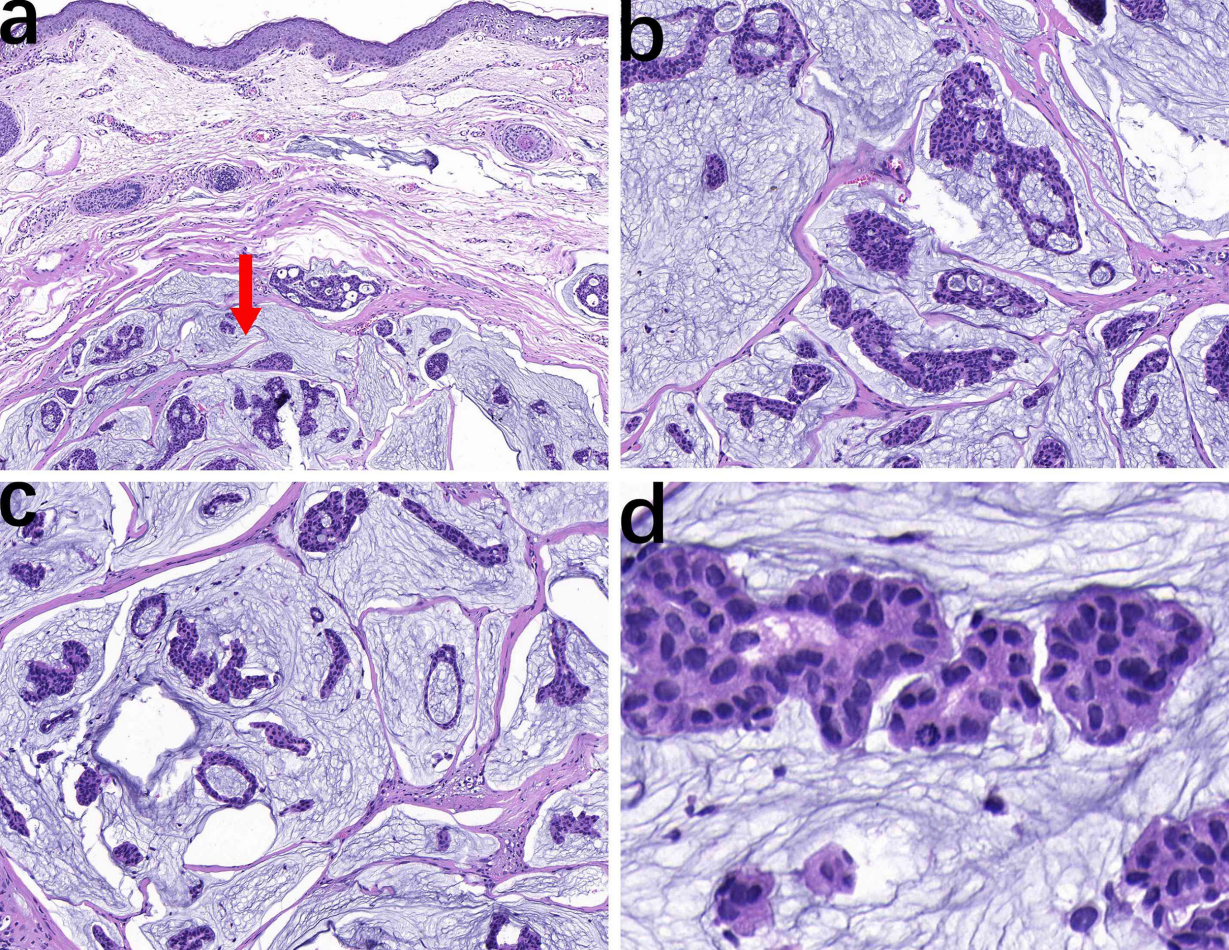
The tumor is located within the dermis with relatively clear boundaries (arrow), and normal dermal tissue is present above (**a**, HE, ×10). Epithelioid cell nests are distributed in pale blue mucinous lakes, separated by delicate collagen-degraded fibrous tissue (**b**, HE, ×20). The tumor cell nests composed of epithelioid cells exhibit small papillary, nested, glandular tubular, and cribriform patterns (**c**, HE, ×20). Cellularly, the cells exhibit low-cuboidal, round, or oval shapes with red-stained cytoplasm, fine and uniform chromatin, focal small nucleoli, and occasional mitotic figures (**d**, HE, ×40).

**Figure 2:**
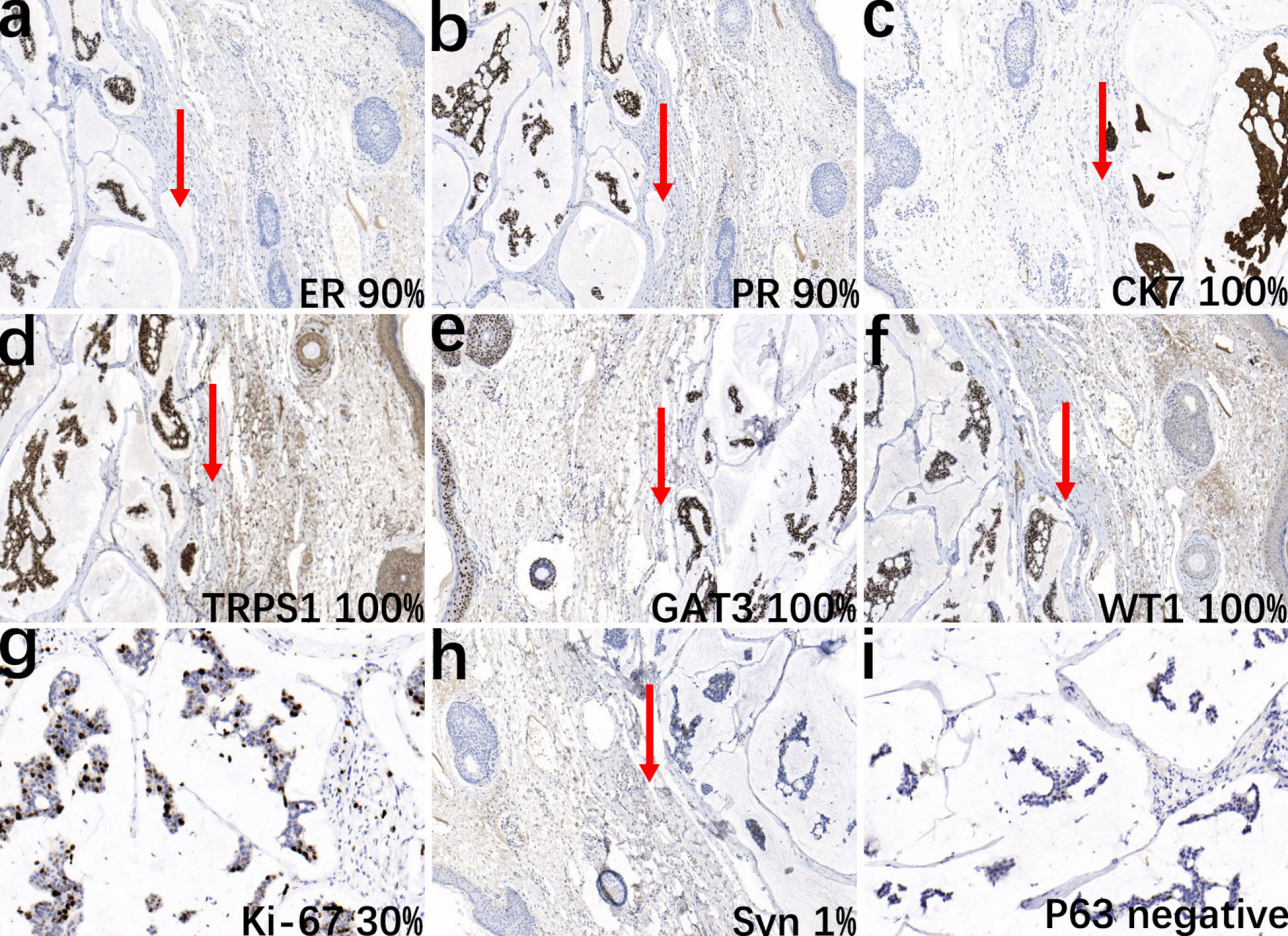
Immunohistochemical analysis of the tumor. The epithelial cell nests show positive staining for ER (**a**, IHC, ×10), PR (**b**, IHC, ×10), CK7 (**c**, IHC, ×10), TRPS1 (**d**, IHC, ×10), GATA3 (**e**, IHC, ×10), WT1 (**f**, IHC, ×10), and Ki-67 (**g**, IHC, ×20), and focal positivity for Syn (**h**, IHC, ×10), while negative for P63 (**i**, IHC, ×20). Arrows indicate the boundary between normal adjacent skin and malignant tissue to facilitate histopathological interpretation.

The pathological examination showed a skin tissue specimen measuring 3 cm x 2 cm x 1.5 cm, with a skin area of 2 cm x 1.5 cm. A mass measuring 2 cm x 1.5 cm x 1.5 cm was found under the epidermis, with a light blue jelly-like cut surface and unclear boundaries. Microscopic observation revealed that the tumor tissue was located within the dermis (**Figure 1a**), with mucous lakes separated by delicate collagenous fibers (**Figure 1b**). Mucinous lakes contained floating clusters of tumor cells composed of epithelioid cells, arranged in various patterns including tightly packed clusters, nests, glandular tubules, and sieve-like networks (**Figure 1c**). The cells were low-cuboidal, round, or oval in shape, with red-stained cytoplasm, finely granular and uniformly distributed chromatin, small nucleoli visible in focal areas, and occasional mitotic figures (**Figure 1d**). In brief, for immunohistochemistry (IHC), tissue samples were fixed in 10% neutral-buffered formalin at room temperature for 12 hours, followed by paraffin embedding and sectioning into continuous 4-μm-thick slices. The paraffin sections were dewaxed and rehydrated at 60°C, then rinsed with PBS for 5 minutes. Antigen retrieval was performed using heat-induced epitope retrieval to expose antigenic sites. To block endogenous peroxidase activity, the sections were incubated with 3% hydrogen peroxide at 37°C for 6 minutes. Subsequently, the primary antibody was applied, and the sections were incubated at 37°C for 30 minutes. A peroxidase-conjugated secondary antibody was then added, followed by a 10-minute incubation at 37°C. For visualization, freshly prepared DAB chromogen was applied and incubated at 37°C for 8 minutes. The sections were then counterstained with hematoxylin for 10 minutes to enhance nuclear contrast. Finally, the slides were dehydrated through graded ethanol solutions, cleared in xylene, and mounted. For quantitative evaluation, positive cells were counted in ten randomly selected high-power fields (400× magnification) by three independent dermatopathologists, and the mean value was calculated. Immunohistochemical staining showed that the tumor cells were strongly positive for ER (90%) (**Figure 2a**), PR (90%) (**Figure 2b**), CK7 (100%) (**Figure 2c**), TRPS1 (100%) (**Figure 2d**), GATA3 (90%) (**Figure 2e**), WT1 (100%) (**Figure 2f**, and Ki-67 (30%) (**Figure 2g**), and focally positive for synaptophysin (Syn) (1%) (**Figure 2h**), while negative for P63 (**Figure 2i**). The final diagnosis was PCMC, supported by the immunophenotypic profile (TRPS1+/GATA3+/ER+/PR+/WT1+/CK7+/P63−) and exclusion of metastatic mucinous carcinoma through clinical and imaging correlation. The patient was discharged 5 days after surgery. The patient did not receive adjuvant therapy after surgery and continued to receive follow-up every 6 months; there has been no evidence of tumor currently after resection.

## Discussion

### Clinicopathological overview of PCMC

PCMC is a rare malignant adnexal neoplasm with indolent biological behavior, first described by Lennox et al. in 1952 as a sweat gland-derived tumor (2). While initially thought to arise from eccrine glands, current consensus favors an apocrine origin (1), consistent with its predilection for regions rich in apocrine glands, such as the head, neck (particularly periorbital areas), and less commonly the axilla, trunk, or genital regions (5, 7). Clinically, PCMC presents as slow-growing, asymptomatic erythematous nodules from 0.5 to 20 cm in diameter, generally exhibiting a favorable prognosis (3, 4). Distant metastasis occurs in only 4%-11% of cases (3), though local recurrence rates are high (6).

### Histopathological subtypes and diagnostic challenges

PCMC can be histologically classified into two subtypes: the pure type, which is characterized by the presence of abundant extracellular mucin accounting for approximately 90% of the tumor volume, and the rare mixed type, which contains infiltrative ductal carcinoma components (8). The mixed type has been reported to mimic syringomatous carcinoma histologically (8), highlighting the diagnostic complexity posed by the tumor’s variant morphologies. The pure-type PCMC in this case demonstrates striking histological overlap with MMC, particularly MBC (9, 10). This morphological resemblance necessitates rigorous clinicopathological correlation and advanced IHC profiling for accurate distinction. The co-expression of GATA3 and TRPS1 observed here highlights both the diagnostic challenges and emerging insights into PCMC’s immunophenotypic landscape, reinforcing the need for biomarker-driven differential diagnosis.

### GATA3: diagnostic utility and limitations

GATA3, a transcription factor central to epithelial differentiation, is widely expressed in breast, urothelial, and cutaneous adnexal tissues (e.g., apocrine glands, hair follicle outer root sheaths) (18). In PCMC, the diffuse and strong positivity of GATA3 (e.g., 90% tumor cell positivity in this case) not only provides critical support for the hypothesized apocrine origin (18, 19) but also underscores its potential dominant role in the tumor’s molecular regulatory network (20). A cohort study of 17 PCMC cases demonstrated consistent high expression of GATA3 across all cases (18), solidifying its status as a core diagnostic marker for PCMC. Notably, GATA3 correlates with hormone receptor (ER/PR) positivity (both 90% in our case), implicating estrogen-response pathways (e.g., ESR1/GREB1) in tumor biology (10, 21). This association rationalizes endocrine therapy (e.g., tamoxifen) for inoperable or high-risk cases (6). The expression of ER and PR makes it challenging to distinguish PCMC from metastatic breast cancer based solely on histopathology and IHC. However, the identification of myoepithelial markers (such as WT1 and P63) may help confirm the tumor’s cutaneous origin. P63 is a marker of epidermal stem cells, involved in the development and regeneration of epithelial cells. The negativity of p63 is often used as a marker of undifferentiated or malignant transformation in tumor cells (22–29). However, GATA3’s expression in MBC (luminal subtypes) and urothelial carcinoma mandates cautious interpretation to avoid misclassifying PCMC as metastatic disease (30).

### TRPS1: beyond mammary specificity

Contrary to earlier assumptions, TRPS1 is not specific to mammary tumors. Embryologic similarities among skin adnexa, breast, and salivary glands underlie TRPS1’s expression in diverse neoplasms, including cutaneous adnexal tumors (13, 17), squamous cell carcinoma (17), and reactive fibroblasts/myofibroblasts in scars (15). While TRPS1 positivity is documented in mammary/extramammary Paget disease (14, 17) and mesenchymal tumors (16), its diagnostic utility in PCMC requires contextual integration. Our findings highlight that TRPS1’s diagnostic value in PCMC lies not in standalone specificity, but in its integration with clinical-pathological context and complementary markers like GATA3. For instance, TRPS1 co-expression with neuroendocrine markers(e.g., INSM1, Syn) strongly supports endocrine mucin-producing sweat gland carcinoma (EMPSGC) (26, 28). Emerging evidence positions PCMC as a precursor to EMPSGC, sharing TRPS1/GATA3/neuroendocrine co-expression (29). Focal Syn positivity in our case aligns with this hypothesis, suggesting neuroendocrine differentiation within mucinous carcinogenesis. Additionally, the expression of neuroendocrine markers such as Syn and chromogranin A (CgA) in some PCMCs suggests neuroendocrine differentiation (10), although the clinical significance of this observation remains unclear. Notably, this case’s Syn positivity hints at potential neuroendocrine features, and GATA3-driven regulation of neuroendocrine transcription factors (e.g., ASCL1/NEUROD1) may interact with GATA5/6, possibly influencing the Hedgehog/Wnt signaling pathways that promote mucin secretion and tumor progression (27, 31). These observations challenge the traditional dichotomy between cutaneous and visceral mucin secretion mechanisms, warranting further investigation.

### Molecular crosstalk and diagnostic strategy

The co-expression of GATA3 and TRPS1 complicates the distinction between PCMC and MBC. Both markers play complementary roles in diagnosing glandular malignancies, as they are involved in regulating key pathways, such as the Wnt/β-catenin pathway, which drives mucin secretion and tumor progression (32). TRPS1 is also implicated in the Hedgehog signaling pathway, and interactions between Hedgehog and Wnt pathways may amplify mucinous differentiation phenotypes (33). In hormone receptor-positive tumors, GATA3 modulates cell proliferation through estrogen-responsive genes (e.g., ESR1 and GREB1) (10, 12), while TRPS1 may similarly influence hormonal signaling in breast cancer. This overlapping pathway involvement suggests that GATA3 and TRPS1 may cooperate to regulate the biological behavior of glandular tumors, although the functional synergy of these factors in PCMC remains an area for future research. To accurately differentiate between PCMC and MBC, a multidimensional diagnostic approach is essential. Key differential approaches include thorough clinical assessment to exclude primary breast lesions via imaging (e.g., mammography, ultrasound), histopathological evaluation to confirm architectural continuity with cutaneous adnexal structures such as sweat gland ducts, and immunohistochemical profiling using a combined panel of markers, particularly TRPS1 (commonly positive in PCMC but often negative in MBC), CK7, and p63 (typically lost in PCMC)—to enhance diagnostic precision (5, 10, 12).

### Differential diagnosis and therapeutic implications

PCMC has a better prognosis compared to skin MMC, so it also needs to be differentiated from the following three types of MMC. Differentiation from MMC of gastrointestinal origin: Gastrointestinal mucinous carcinoma is known as a “dirty cancer.” Combining clinical endoscopic findings and immunohistochemical staining results, which are usually negative for CDX2 and CK20 but positive for CK7, can generally exclude this possibility (34). Differentiation from MMC of prostate and lung origin: Combining medical history, physical examination, and imaging studies can differentiate tumors of prostate and lung origin. Negative staining for PSA and NKX3.1 can exclude prostate origin, while negative staining for TTF-1 and NapsinA can exclude lung origin. Since this case of PCMC has neuroendocrine expression, it also needs to be differentiated from Merkel cell carcinoma (MCC) of the skin (35): MCC is a highly aggressive neuroendocrine carcinoma of the skin with high malignancy. The tumor cells grow in characteristic trabecular, solid, or nodular patterns, with vacuolated nuclei and typical granular chromatin that appears dusty (36). Immunohistochemical staining for CK20 shows a perinuclear dot-like pattern. In summary, PCMC and skin metastatic mucinous carcinoma are histologically very similar, especially when using TRPS1 and GATA3 for differential diagnosis between PCMC and breast MBC. It is necessary to closely integrate clinical information and exclude the possibility of metastasis from the breast, prostate, lung, and gastrointestinal tract. Our findings highlight that TRPS1’s diagnostic value in PCMC lies not in standalone specificity, but in its integration with clinical-pathological context and complementary markers like GATA3.

PCMC, a malignant primary cutaneous adnexal neoplasm, exhibits approximately 11% recurrence and 11% metastatic cases (lung/bone) with 2 fatalities (35). It is resistant to radiotherapy/chemotherapy and primarily managed with surgical resection (5, 37). Endocrine therapy is adjuvant due to ER/PR expression. PCMC has a superior prognosis versus cutaneous MMC, underscoring the need for early diagnosis and differential accuracy. A multimodal analysis (clinical, imaging, histopathology, IHC) is pivotal for diagnosis, progression assessment, and prognostic evaluation.

## Conclusion

Our findings highlight that TRPS1’s diagnostic value in PCMC lies not in standalone specificity, but in its integration with clinical-pathological context and complementary markers like GATA3. Comprehensive analysis combining clinical, imaging, histopathological, and immunohistochemical data remains indispensable for accurate diagnosis and prognostication. Future studies should explore the functional interplay between GATA3, TRPS1, and neuroendocrine pathways to refine therapeutic strategies for this enigmatic malignancy.

## Data Availability

The datasets generated for this study are available from the corresponding author on reasonable request.
